# Comparison of clinical characteristics and disease burden between early- and late-onset type 2 diabetes patients: a population-based cohort study

**DOI:** 10.1186/s12889-023-17280-5

**Published:** 2023-12-04

**Authors:** Mingqi Wang, Yifei He, Qiao He, Fusheng Di, Kang Zou, Wen Wang, Xin Sun

**Affiliations:** 1 Chinese Evidence-based Medicine Center and Cochrane China Center, West China Hospital, Sichuan University, 37 Guo Xue Xiang, Chengdu, 610041 Sichuan China; 2 NMPA Key Laboratory for Real World Data Research and Evaluation in Hainan, West China Hospital, Sichuan University, 37 Guo Xue Xiang, Chengdu, 610041 China; 3 Sichuan Center of Technology Innovation for Real World Data, Chengdu, 610041 China; 4 Department of Endocrinology, Tianjin Third Central Hospital, Tianjin, 300000 China

**Keywords:** New-onset type 2 Diabetes, Population-based cohort study, Comorbid Disease development

## Abstract

**Background:**

The clinical characteristics of early-onset type 2 diabetes (T2D) patients are not fully understood. To address this gap, we conducted a cohort study to evaluate clinical characteristics and disease burden in the new-onset T2D population, especially regarding the progression of diseases.

**Methods:**

This cohort study was conducted using a population-based database. Patients who were diagnosed with T2D were identified from the database and were classified into early- (age < 40) and late-onset (age ≥ 40) groups. A descriptive analysis was performed to compare clinical characteristics and disease burden between early- and late-onset T2D patients. The progression of disease was compared using Kaplan‒Meier analysis.

**Results:**

A total of 652,290 type 2 diabetic patients were included. Of those, 21,347 were early-onset patients, and 300,676 were late-onset patients. Early-onset T2D patients had poorer glycemic control than late-onset T2D patients, especially at the onset of T2D (HbA1c: 9.3 [7.5, 10.9] for early-onset vs. 7.7 [6.8, 9.2] for late-onset, *P* < 0.001; random blood glucose: 10.9 [8.0, 14.3] for early-onset vs. 8.8 [6.9, 11.8] for late-onset, *P* < 0.001). Insulin was more often prescribed for early-onset patients (15.2%) than for late-onset patients (14.8%). Hypertension (163.0 [28.0, 611.0] days) and hyperlipidemia (114.0 [19.0, 537.0] days) progressed more rapidly among early-onset patients, while more late-onset patients developed hypertension (72.7% vs. 60.1%, *P* < 0.001), hyperlipidemia (65.4% vs. 51.0%, *P* < 0.001), cardiovascular diseases (66.0% vs. 26.7%, *P* < 0.001) and chronic kidney diseases (5.5% vs. 2.1%, *P* < 0.001) than early-onset patients.

**Conclusions:**

Our study results indicate that patients with newly diagnosed early-onset T2D had earlier comorbidities of hypertension and hyperlipidemia. Both clinical characteristics and treatment patterns suggest that the degree of metabolic disturbance is more severe in patients with early-onset type 2 diabetes. This highlights the importance of promoting healthy diets or lifestyles to prevent T2D onset in young adults.

**Supplementary Information:**

The online version contains supplementary material available at 10.1186/s12889-023-17280-5.

## Introduction

The prevalence of diabetes in China has increased dramatically from less than 1% in 1980 to approximately 12.4% in 2018 [1]. Type 2 diabetes (T2D) has the highest prevalence, accounting for approximately 90% of all patients [2, 3]. Although T2D is generally thought to be a disease of the middle and elderly, there has been a notable increase in the number of young adults affected by T2D (defined as early-onset T2D in adults aged < 40 years [4, 5] in recent years [6–9]. The results of a study showed that the number of patients with T2D increased by 31% between 2001 and 2009 among American teenagers aged 10 to 19 [10]. In the UK, between 1996 and 2000 and 2006–2010, the standardized incidence ratio (per 100,000 people) of newly diagnosed T2D among people under the age of 40 more than doubled from 217 to 598 [11]. Diabetes prevalence increased from 3.2% in 2010 to 5.9% in 2013 in China’s population younger than 40 [12, 13]. Early-onset T2D is a growing concern in all countries, with the proportion of young T2D patients increasing [14, 15].

Although the prevalence of early-onset T2D has increased over time, the clinical features, treatment patterns, and disease burden are still not fully understood. As prior studies show, that the most common comorbidities of T2D is that hypertension and hyperlipidemia [16, 17], but disease progression are still not well understood in early-onset and late-onset T2D. Meanwhile, some of the associated metabolic characteristics remain controversial, such as BMI and low-density lipoprotein cholesterol (LDL-C) levels [18, 19], with studies from different countries reporting conflicting results [13]. In addition, the medication regimen of T2D patients varies widely by region. For instance, an observational study involving 12 countries showed that the proportion of patients with T2D using the same hypoglycemic agent varied considerably by country [20]. Finally, it is important to understand the burden of disease, and although there are some studies on the burden of disease in T2D and its complications [21, 22], there have been few relevant studies in China in recent years.

In addition, complications are the most important factors affecting patient prognosis in both early-onset and late-onset T2D. Cardiovascular disease (CVD) and chronic kidney disease (CKD) are the leading causes of death in T2D patients. However, the majority of previous investigations have focused on the prevalence and risk of complications related to early-onset T2D [23, 24]. The progression of complications and comorbidities may differ between early-onset and late-onset T2D due to their different clinical features. Early-onset T2D may have earlier onset of complications and comorbidities due to more severe metabolic disorders, and understanding the progression is crucial for better clinical management of patients.

In view of the above issues, we conducted a cohort study based on a population-based database to compare the clinical characteristics, treatment pattern and progression of disease between early-onset and late-onset T2D populations.

## Research design and methods

This cohort study was conducted based on a population-based database. This study was approved by the Ethical Committee for Clinical Research (SECCR/2021-81-01). All data were collected for administrative and clinical purposes without specific goals, and all patients’ private information was deidentified; therefore, consent was waived.

### Data source

This study was conducted based on the Tianjin regional healthcare database, which covers over 15 million patients’ individual-level healthcare information from approximately 300 tertiary, secondary and community hospitals in Tianjin up to December 2019. The database includes detailed information on demographics, diagnosis, prescriptions, operations, tests, examinations and outpatient and hospitalization costs. By using deidentified identification numbers, data from different sources can be linked to each patient.

### Study population

Patients who diagnosed as diabetes between January 1, 2015 and December 30, 2019 were identified using the International Classification of Disease (ICD) code from the database. We also extracted data regarding visit information before 2015 for each of the patients.

T2D patients were included according to the following rules:

1. patients with at least 1 discharged diagnosis or 2 outpatient diagnoses of T2D;
2. patients with a diagnosis of diabetes could be defined as having T2D only when they met the following criteria: at least two records of outpatient visits involving an oral hypoglycemic drug (OHA) prescription only or one record of hospitalization with OHA prescribed but no insulin; meanwhile, patients should be older than 30 years old on the first date of diabetes diagnosis.

Patients were excluded when they met the following criteria:

1. patients who diagnosed as type 1 diabetes, gestational diabetes or other types of diabetes (mitochondrial diabetes, etc.);
2. patient of non- Chinese nationality.

New-onset T2D patients were defined as patients who had at least one visit without diabetes diagnosis prior to the first T2D diagnosis. The T2D onset date was defined as the first diagnosis date of diabetes. Then, the new-onset T2D patients were classified into groups of early-onset patients ( < = 40 years old) and late-onset patients (> 40 years old). The new- onset of comorbid diseases as the comorbid disease onset date was not earlier than the T2D onset date. Onset date was defined as the first inpatient diagnosis or the earlier date of two consecutive outpatient diagnoses of comorbid diseases.

### Statistical analysis

Descriptive analysis was performed to summarize and compare the demographic and clinical characteristics, disease burden and progression of comorbidities/complications between early- and late-onset diabetic patients. For the results during the T2D period, we analyzed the average values of HbA1c and random glucose for each patient calculating during their T2D period. In addition, values from the last visit during the T2D period were also used for analysis. Follow-up depended on the duration of routine care patients received. Continuous data were summarized through means, standard deviations, medians and quantiles, whereas categorical data were summarized as counts and proportions. The two-sample t test or Mann‒Whitney U test was used to compare the mean or median, and the chi-square test was used to compare groups.

Kaplan‒Meier survival analysis was conducted to demonstrate the progression of comorbidities and complications among all new-onset T2D patients. Kaplan‒Meier survival analysis was also performed for patients during the 180 days and 365 days after T2D onset to compare the short-term progression of comorbidities/complications. R version 4.1.2 was used to extract, clean and analyze data, and statistical significance was accepted at *P* < 0.05.

## Results

**Fig. 1:**
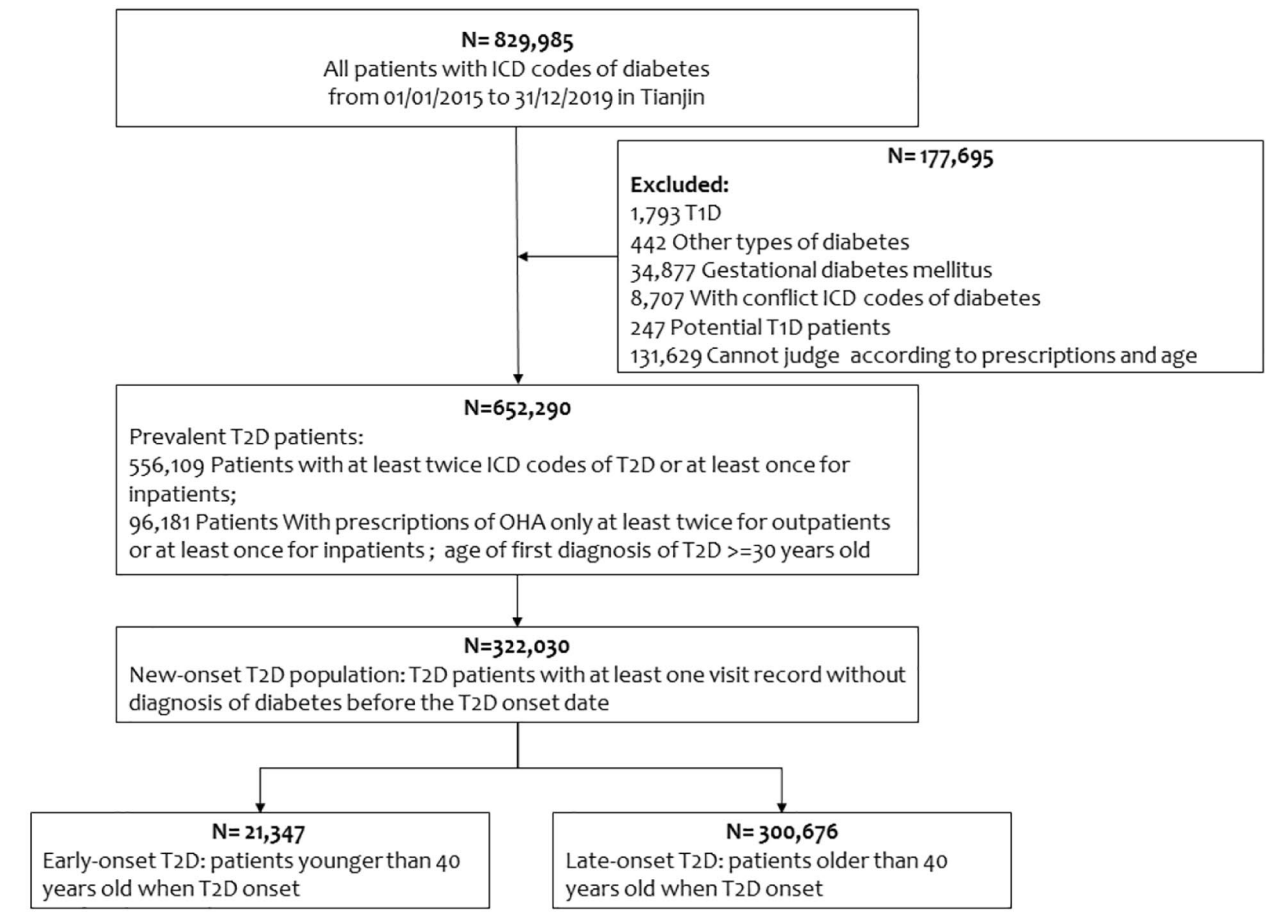
Workflow for selection of new-onset T2D patients

There were 829,985 patients of 62 secondary and tertiary hospitals identified as having diabetes from the database; as Fig. 1 shows, there were 1793 patients with type 1 diabetes, 247 potential diabetic patients, 34,877 patients with gestational diabetes, 8707 patients with conflicting diagnoses, and 131,629 patients for whom the diabetes type could not be determined. Finally, 652,290 (78.6%) patients were identified as having T2D, and 322,030 patients were identified as having new-onset T2D. Among these new-onset T2D patients, 21,347 (6.6%) patients had early-onset disease, and 300,676 (93.4%) patients had late-onset disease (Fig. 1).

### Clinical characteristics

More male patients developed T2D in the early-onset population than in the late-onset population (62.7% vs. 51.5%, *P* < 0.001). Most T2D patients were in the age of 52 to 68 years. The median age of late-onset patients was 61.8 years, while the median age was 35.0 years old for early-onset patients.

As shown in Tables 1 and 2, early-onset patients had worse glycemic status than late-onset patients during the T2D course (HbA1c: 7.9 [6.8, 9.6] vs. 7.4 [6.7, 8.6], *P* < 0.001; random blood glucose: 8.3 [6.8, 10.7] vs. 8.1 [6.8, 10.0], *P* < 0.001), and glycemic status was worse at the onset of T2D (HbA1c: 9.3 [7.5, 10.9] vs. 7.7 [6.8, 9.2], *P* < 0.001; random blood glucose: 10.9 [8.0, 14.3] vs. 8.8 [6.9, 11.8], *P* < 0.001). The same tendency was observed in the results of other measurements. Values of renal function measurements were better among early-onset patients (eGFR: 102.8 [82.1, 120.0] vs. 76.0 [58.7, 94.8], *P* < 0.001; serum creatine: 64.3 [53.5, 75.0] vs. 65.7 [55.0, 79.0], *P* < 0.001; MALB: 13.8 [5.8, 41.0] vs. 11.2 [5.0, 33.5], *P* < 0.001) in the course of T2D, while values of blood lipid measurements were better among late-onset patients (LDL: 3.1 [2.6, 3.6] vs. 3.0 [2.4, 3.6], *P* < 0.001; TG: 2.0 [1.4, 3.0] vs. 1.5 [1.1, 2.1], *P* < 0.001; TC: 4.9 [4.3, 5.6] vs. 4.8 [4.0, 5.5], *P* < 0.001).

**Table 1:** Clinical characteristics of T2D patients during period*

	T2D (N = 652,290)	New-onset T2D (N = 322,030)		*P* value
		Early-onset T2D (N = 21,347)	Late-onset T2D (N = 300,676)	
**Baseline characteristics**				
Age on first diagnosis date (M, IQR)	60.5[52.0, 68.1]	35.0 [32.0, 37.5]	61.8 [55.0, 69.1]	< 0.001
Male (N, %)	348,381(53.4)	13,383 (62.7)	154,834 (51.5)	< 0.001
**Laboratory measurements (M, IQR)**				
HbA1c (%)	7.7 [6.8, 9.0]	7.9 [6.8, 9.6]	7.4 [6.7, 8.6]	< 0.001
Random blood glucose (mmol/L)	8.5 [7.0, 10.6]	8.3 [6.8, 10.7]	8.1 [6.8, 10.0]	< 0.001
Serum creatinine (umol/L)	65.8 [54.8, 79.1]	64.3 [53.5, 75.0]	65.7 [55.0, 79.0]	< 0.001
eGFR (ml/min/1.73m^2^)	76.0 [57.4, 96.0]	102.8 [82.1, 120.0]	76.0 [58.7, 94.8]	< 0.001
**Number of patients tested MALB**	97,736	2977	37,334	
MALB(g/24 h)	14.5 [7.4, 45.5]	13.8 [5.8, 41.0]	11.2 [5.0, 33.5]	< 0.001
*Microalbuminuria (n, %)*	3,816 (3.9)	122 (4.1)	1,351 (3.6)	0.012
*Macroalbuminuria (n, %)*	66,449 (68.0)	1,924 (64.6)	25,783 (69.1)	0.028
HDL (mmol/L)	1.1 [0.9, 1.3]	1.0 [0.8, 1.1]	1.0 [0.9, 1.2]	< 0.001
LDL (mmol/L)	3.0 [2.4, 3.7]	3.1 [2.6, 3.6]	3.0 [2.4, 3.6]	< 0.001
TG (mmol/L)	1.5 [1.1, 2.2]	2.0 [1.4, 3.0]	1.5 [1.1, 2.1]	< 0.001
TC (mmol/L)	4.8[4.0, 5.6]	4.9 [4.3, 5.6]	4.8 [4.0, 5.5]	< 0.001
**Treatment pattern (N, %)**				
Anti-hypertension	352,736 (54.1)	7,326 (34.3)	167,582 (55.7)	< 0.001
Anti-hyperlipidaemia	261,606 (40.1)	6,094 (28.5)	116,890 (38.9)	< 0.001
Anti-platelets	290,832 (44.6)	3,533 (16.6)	137,575 (45.8)	< 0.001
Insulin	297,026 (45.5)	7,703 (36.1)	126,359 (42.0)	< 0.001
Oral hypoglycemic drugs	474,387 (72.7)	15,527 (72.7)	218,657 (72.7)	0.97
*Sulfonylureas*	179,460 (27.5)	5,479 (25.7)	81,920 (27.2)	< 0.001
*Metformin*	311,612 (47.8)	11,667 (54.7)	139,333 (46.3)	< 0.001
*Incretin (DDP-4i, GLP-1)*	104,773 (16.1)	4,058 (19.0)	45,871 (15.3)	< 0.001
*SGLT-2*	2,139 (0.3)	139 (0.7)	825 (0.3)	< 0.001
*α- glucosidase inhibitor*	349,191 (53.5)	9,072 (42.5)	162,582 (54.1)	< 0.001
*Thiazolidinediones*	88,658 (13.6)	4,078 (19.1)	39,509 (13.1)	< 0.001
*Compound*	1,976 (0.3)	142 (0.7)	545 (0.2)	< 0.001
*Others*	153,578 (23.5)	3,444 (16.1)	72,615 (24.2)	< 0.001
**Comorbidities/complications**				
Cardiovascular disease	204,257 (31.3)	5,698 (26.7)	198,559 (66.0)	< 0.001
Chronic kidney disease	17,015 (2.6)	450 (2.1)	16,565 (5.5)	< 0.001
Hypertension	180,161 (27.6)	7,289 (34.1)	172,872(57.5)	< 0.001
Hyperlipidemia	209,074 (32.1)	10,866 (50.9)	198,208 (65.9)	< 0.001

*The duration was defined as from the onset of T2D to the end of follow-up, follow-up depends on the duration of patients’ routine care in the database

**Table 2:** Clinical characteristics of new-onset T2D patients when onset

	Early-onset T2D (N = 21,347)	Late-onset T2D (N = 300,676)	*P* value
**Baseline characteristics**			
Age on first diagnosis date (M, IQR)	35.0 [32.0, 37.5]	61.8 [55.0, 69.1]	< 0.001
Male (N, %)	13,383 (62.7)	154,834 (51.5)	< 0.001
**Laboratory measurements (M, IQR)**			
HbA1c (%)	9.3 [7.5, 10.9]	7.7 [6.8, 9.2]	< 0.001
Random blood glucose (mmol/L)	10.9 [8.0, 14.3]	8.8 [6.9, 11.8]	< 0.001
Serum creatinine (umol/L)	62.4 [51.6, 73.7]	65.0 [54.0, 78.8]	< 0.001
eGFR (ml/min/1.73m^2^)	117.0 [101.7, 124.9]	84.0 [63.5, 99.7]	< 0.001
**Number of patients tested MALB**	10,995	121,547	
MALB(g/24 h)	17.9 [7.6, 68.0]	10.0 [4.3, 31.6]	< 0.001
*Microalbuminuria (n, %)*	23 (0.2)	120(0.1)	< 0.001
*Macroalbuminuria (n, %)*	370 (3.4)	3,434 (2.8)	< 0.001
HDL (mmol/L)	1.0 [0.8, 1.2]	1.1 [0.9, 1.3]	< 0.001
LDL (mmol/L)	3.2 [2.6, 3.8]	3.1 [2.4, 3.7]	< 0.001
TG (mmol/L)	2.2 [1.5, 3.6]	1.6 [1.1, 2.2]	< 0.001
TC (mmol/L)	5.1 [4.4, 5.9]	4.8 [4.0, 5.6]	< 0.001
**Treatment pattern (N, %)**			
Anti-hypertension	2,464 (11.5)	53,054 (17.6)	< 0.001
Anti-hyperlipidaemia	1,842 (8.6)	32,635 (10.9)	< 0.001
Anti-platelets	814 (3.8)	34,605 (11.5)	< 0.001
Insulin	3,243 (15.2)	44,461 (14.8)	0.11
Oral hypoglycemic drugs	2,087 (9.8)	27,371 (9.1)	0.001
*Sulfonylureas*	4,747 (22.2)	47,225 (15.7)	< 0.001
*Metformin*	853 (4.0)	5,958 (2.0)	< 0.001
*Incretin (DDP-4i, GLP-1)*	5 (0.0)	50 (0.0)	0.643
*SGLT-2*	2,087 (9.8)	27,371 (9.1)	0.001
*α-glucosidase inhibitor*	3,486 (16.3)	60,774 (20.2)	< 0.001
*Thiazolidinediones*	1,304 (6.1)	11,781 (3.9)	< 0.001
*Compound*	27 (0.1)	97 (0.0)	< 0.001
*Others*	1,303 (6.1)	22,587 (7.5)	< 0.001
**Comorbidities/complications**			
Cardiovascular disease	1,398 (6.5)	77,834 (25.9)	< 0.001
Chronic kidney disease	61 (0.3)	1,483 (0.5)	< 0.001
Hypertension	2,419 (11.3)	77,181 (25.7)	< 0.001
Hyperlipidemia	3,253 (15.2)	84,256 (28.0)	< 0.001

The proportion of patients with CVD (66.0% vs. 26.7%, *P* < 0.001), CKD (5.5% vs. 2.1%, *P* < 0.001), hypertension (34.1% vs. 57.5%, *P* < 0.001) and hyperlipidemia (50.9% vs. 65.9%, *P* < 0.001) was higher in late-onset T2D patients than in early-onset patients during the entire course of T2D. The proportion of the new-onset population who developed CKD was low among all new-onset populations (1.8% in early-onset patients and 5.0% in late-onset patients). Male patients accounted for a major proportion of the population who developed CVD (67.0% vs. 50.8%, *P* < 0.001), CKD (68.4% vs. 53.5%, *P* < 0.001), hypertension (72.7% vs. 60.1%, *P* < 0.001) or hyperlipidemia (65.4% vs. 51.0%, *P* < 0.001), especially in early-onset patients.

### Treatment pattern

The drugs used most frequently during the T2D course were OHAs, followed by insulin. And OHAs were seldom prescribed at the onset of T2D (9.8% for early-onset patients, 9.1% for late-onset patients), and sulfonylureas were the most prescribed (22.2% for early-onset patients, 15.7% for late-onset patients). Insulin was more often prescribed (15.2% for early-onset patients, 14.8% for late-onset patients). During the T2D course, metformin (54.7% vs. 46.3%, *P* < 0.001) and thiazolidinediones (19.1% vs. 13.1%, *P* < 0.001) were prescribed more frequently among early-onset patients, and insulin was prescribed more frequently for late-onset T2D patients (42.0% vs. 36.1%, *P* < 0.001).

### Disease burden

As Table 3 shows, late-onset patients had more records of visits and longer lengths of stay, while most patients visiting the outpatient department were in the group of early-onset patients. All costs of late-onset patients were higher than those of early-onset patients during the T2D period. Late-onset patients sought outpatient treatment more frequently than early-onset patients (18.0 [5.0, 51.0] ¥ vs. 10.0 [4.0, 26.0] ¥, *P* < 0.001), which also resulted in a greater total cost for the late-onset patients (1^1^,667.0 [2,988.0, 34,200.5] ¥ vs. 3,468.0 [1,190.0, 11,054.0] ¥). The gap of hospitalization cost (8,764.0 [6,292.0, 13,675.0] ¥ vs. 11,605.0 [7,815.0, 21,421.0] ¥) or outpatient cost (257.0 [137.0, 452.0] ¥ vs. 308.0 [165.0, 523.0] ¥) per visit was not as huge as the difference in total cost between early-onset and late-onset patients.

**Table 3:** Disease burden of diabetic patients

	T2D (N = 652,290)	New-onset T2D		*P* value
		Early-onset T2D (N = 21,347)	Late-onset T2D (N = 300,676)	
**Onset**				
Number of outpatients	-	17,720 (82.4)	209,846 (69.5)	< 0.001
Number of inpatient	-	3,782 (17.6)	92,103 (30.5)	
Length of stay (days)	-	9.0 [7.0, 12.0]	9.5 [6.5, 13.0]	< 0.001
Total cost (¥)	-			
Outpatient	-	302.0 [153.0, 524.0]	328.0 [172.0, 536.0]	< 0.001
Inpatient	-	9,421.0 [6,437.5, 15,926.5]	12,243.0 [8,124.0, 22,843.0]	< 0.001
**During period**				
Total admissions/patient	12.0 [3.0, 43.0]	9.0 [3.0, 24.0]	12.0 [4.0, 43.0]	< 0.001
Outpatient	18.0 [5.0, 53.0]	10.0 [4.0, 26.0]	18.0 [5.0, 51.0]	< 0.001
Inpatient	1.0 [1.0, 2.0]	1.0 [1.0, 2.0]	1.0 [1.0, 2.0]	< 0.001
Length of stay /patient (days)	9.3 [6.5, 12.7]	9.0 [7.0, 12.0]	9.5 [6.5, 13.0]	< 0.001
Length of stay /visit (days)	10.0 [6.0, 14.0]	9.0 [6.0, 13.0]	9.0 [6.0, 14.0]	< 0.001
Total cost/patients (¥)	11,523.0 [3,319.0, 32,785.0]	3,468.0 [1,190.0, 11,054.0]	11,667.0 [2,988.0, 34,200.5]	< 0.001
Outpatient/visit	402.0 [240.0, 590.0]	344.0 [195.0, 544.0]	390.0 [232.0, 576.0]	< 0.001
Inpatient/visit	11,351.0 [7,526.0, 19,884.0]	9,288.5 [6,374.8, 15,643.0]	11,831.0 [7,874.0, 21,368.0]	< 0.001
**Cost of last visit (¥)**				
Outpatient	313.0 [168.0, 530.0]	257.0 [137.0, 452.0]	308.0 [165.0, 523.0]	< 0.001
Inpatient	11,217.0 [7,498.0, 20,255.0]	8,764.0 [6,292.0, 13,675.0]	11,605.0 [7,815.0, 21,421.0]	< 0.001

#### Progression of comorbidities and complications

**Fig. 2:**
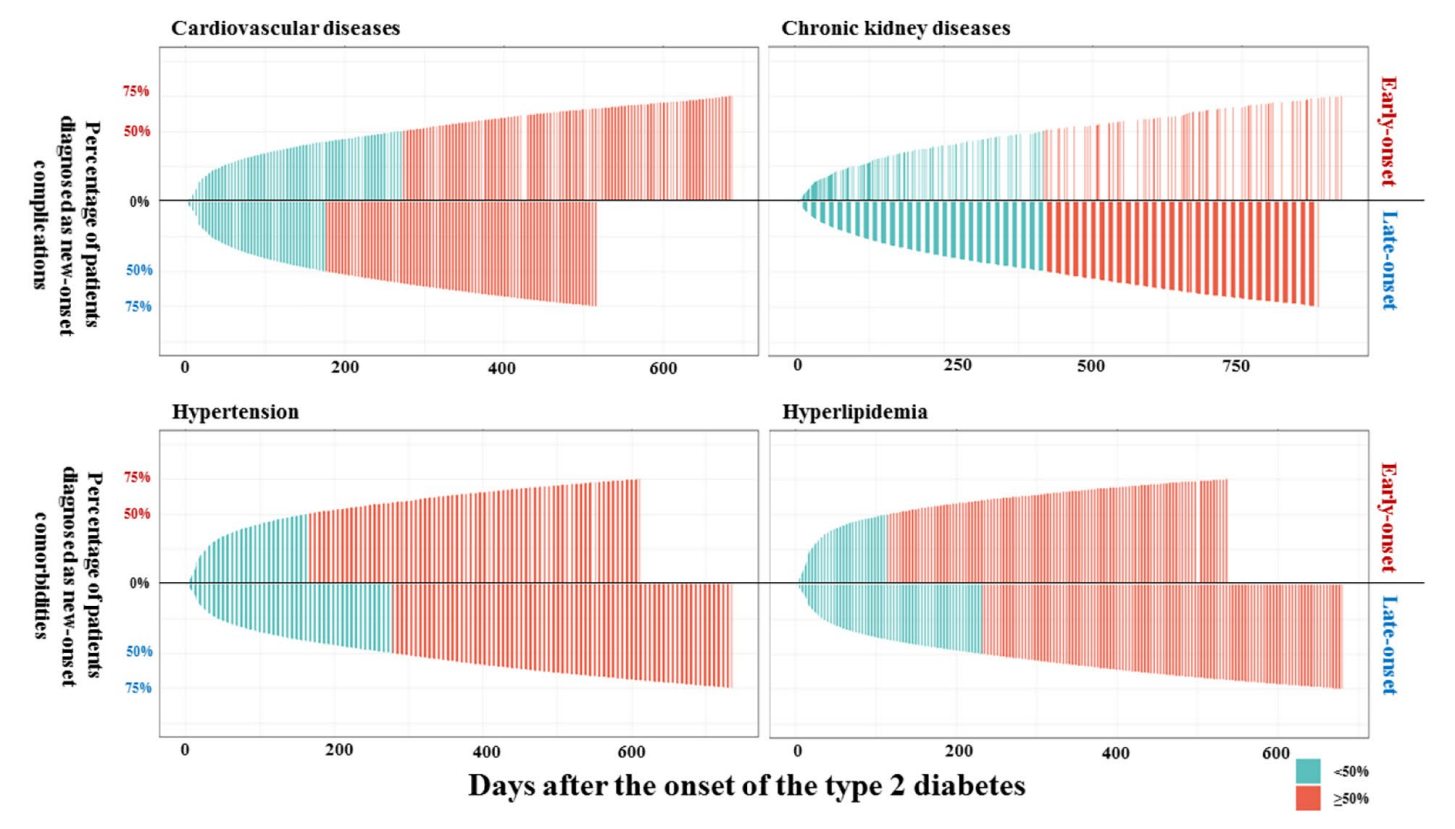
Progression of new-onset complications/comorbidities among new-onset type 2 diabetes patients with comorbid diseases We demonstrated the progression of new-onset comorbid diseases among new-onset type 2 diabetes patients. The height of histogram means the cumulative incidence of comorbid diseases among new-onset patients with comorbidities or complications. As the plot shows, proportion of hypertension and hyperlipidemia reached to 50% and 75% more rapidly among early-onset patients. The last green bar and last red bars represent the rate of comorbidities/complications progression

**Fig. 3:**
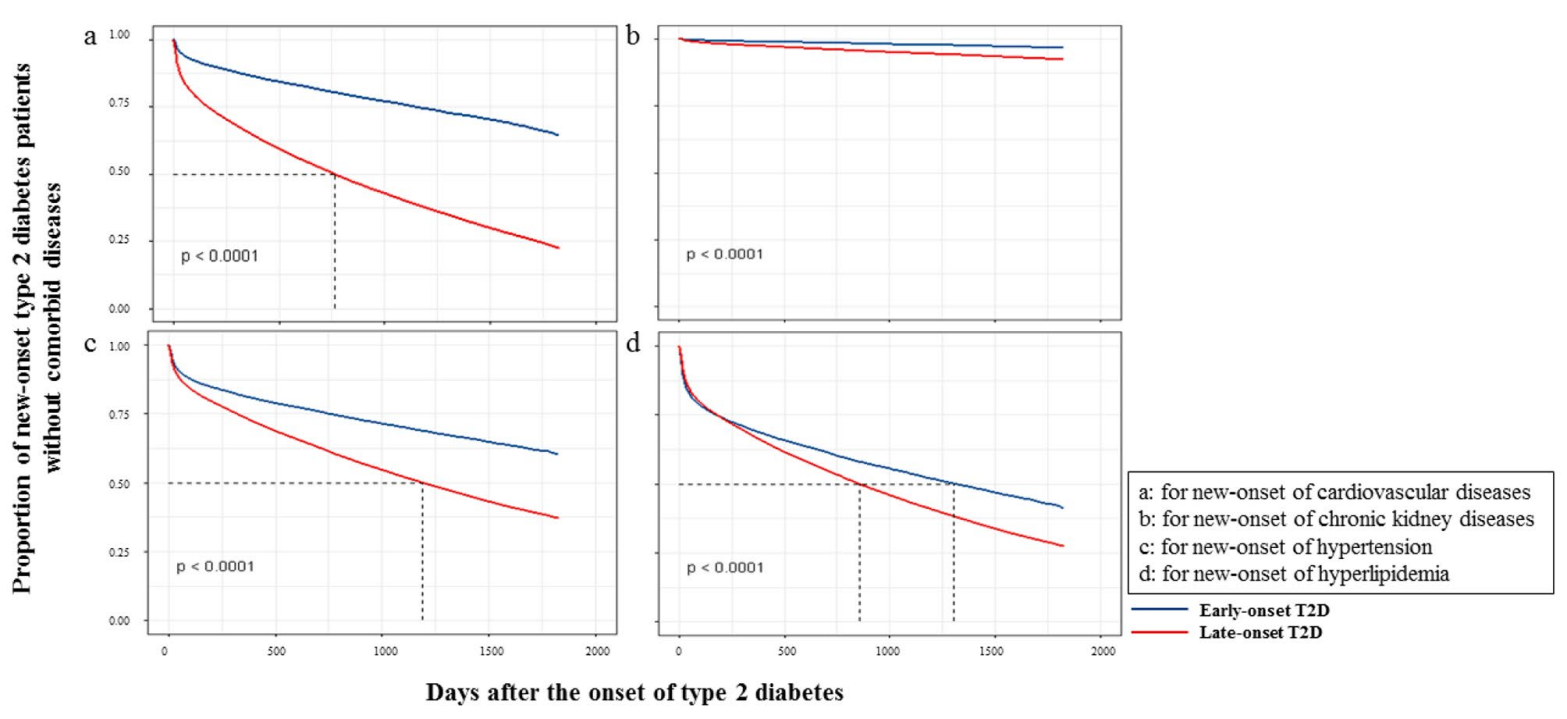
Cumulative hazard of developing complications/comorbidities among new-onset type 2 diabetes patients A Kaplan Meier analysis was conducted to analyze the development of comorbid diseases among all new-onset type 2 diabetes (T2D) patients during the study period. As the plot shows, the prevalence of all comorbid diseases is higher among late-onset type 2 diabetes patients, but the speed of progression differs

As Fig. 2; Table 4 show, for new-onset T2D patients with comorbid diseases, CVD progressed more rapidly in late-onset patients (227.0 [38.0, 651.0] days vs. 301.0 [51.0, 750.0] days, *P* < 0.001), while no significant difference was observed for CKD progression (428.0 [109.0, 890.0] days vs. 423.0 [111.0, 931.0] days, *P* = 0.724). Hypertension (163.0 [28.0, 611.0] days vs. 278.0 [43.0, 735.0] days, *P* < 0.001) and hyperlipidemia (114.0 [19.0, 537.0] days vs. 232.0 [34.0, 680.0] days, *P* < 0.001) progressed more rapidly among the early-onset population. As Fig. 3 and Supplementary Fig. 1 show, during the first 180 days among all new-onset T2D patients, the progression of hyperlipidemia was more rapid for early-onset patients, and hypertension seemed to be similar between early-onset and late-onset T2D patients.

**Table 4:** Development of complications/comorbidities among new-onset T2D patients

Complications	Early-onset T2D (N = 21,347)	Late-onset T2D (N = 300,676)	*P*-Value
**New CVD onset**			
Number of patients (N)	4,300(20.1)	120,725(40.2)	< 0.001
Male (N, %)	2,883 (67.0)	61,375 (50.8)	< 0.001
Days after diabetes onset (M, IQR)	301.0 [51.0, 750.0]	227.0 [38.0, 651.0]	< 0.001
**New CKD onset**			
Number of patients (N)	389(1.8)	15,082(5.0)	< 0.001
Male (N, %)	266 (68.4)	8,063 (53.5)	< 0.001
Days after diabetes onset (M, IQR)	423.0 [111.0, 931.0]	428.0 [109.0, 890.0]	0.724
**New Hypertension onset**			
Number of patients (N)	4,870 (22.8)	95,691 (31.8)	< 0.001
Male (N, %)	3,539 (72.7)	57,495 (60.1)	< 0.001
Days after diabetes onset (M, IQR)	163.0 [28.0, 611.0]	278.0 [43.0, 735.0]	< 0.001
**New Hyperlipidemia onset**			
Number of patients (N)	7,613(35.7)	113,952(37.9)	< 0.001
Male (N, %)	4,981 (65.4)	58,121 (51.0)	< 0.001
Days after diabetes onset (M, IQR)	114.0 [19.0, 537.0]	232.0 [34.0, 680.0]	< 0.001

## Discussion

### Main findings

Our large-scale population-based study revealed that early-onset T2D patients experienced more rapid progression of hypertension and hyperlipidemia than late-onset patients. Concerning general clinical characteristics, early-onset T2D patients had worse metabolic indicators, implying that early-onset patients are more likely to have a metabolic disorder or even metabolic syndrome (MetS).

Late-onset T2D patients were treated more frequently, whereas insulin was prescribed more frequently for early-onset T2D patients at the onset of T2D. Furthermore, we discovered that of the OHAs, metformin was used the most among early-onset T2D patients—more than among late-onset patients. Previous studies have found that high levels of triglycerides contribute to insulin-resistance (IR) and the development of diabetes [25, 26]. The most often prescribed medication to improve insulin sensitivity in IR situations is metformin [27]. Our study found that early-onset T2D had higher TG levels, suggesting that their IR may be more pronounced hence more proportional use of metformin.

Late-onset T2D suffer greater rates of complications, longer hospital stays, and higher costs. Most current studies on disease burden focus on the cost of hospitalization and find that late-onset T2D costs more [28, 29], and our study yielded the same results. Meanwhile, we compared the length of hospitalization and the proportion of comorbidities and complications; both were higher in late-onset patients than in early-onset patients, which is consistent with the costs and reflects the fact that the disease burden in late-onset T2D is still enormous.

### Comparison with other studies

The results of previous studies, as well as ours, have indicated a more severe metabolic disorder in early-onset type 2 diabetes, both in general characteristics and treatment patterns. A cohort study conducted in Sweden described the cumulative incidence and risk factors for comorbid diseases for newly diagnosed T2D patients, but they didn’t evaluate the progression of hypertension or hyperlipidemia and the difference between early-onset and late-onset T2D [30]. One study showed that patients with early-onset diabetes are more likely to have combined hyperlipidemia [31], and another study of 1,421 cases revealed that patients with early-onset T2D had higher triglycerides (TG) and glycated hemoglobin (HbA1c) [32], all of which suggest that patients with early-onset diabetes are clinically characterized by metabolic disorders. In our large sample study, we found that patients with early-onset T2D develop hyperlipidemia and hypertension more rapidly, which is also consistent with metabolic disorders. At baseline, triglyceride concentrations were higher in patients with early-onset T2D than in those with late-onset T2D, according to data from the large JADE registry [18]. Our study had similar results. This suggests that patients with early-onset T2D have higher overall lipid levels, many are borderline hyperlipidemia, and may progress to hyperlipidemia more quickly in the future, which explains our main findings. A study of the risk trajectory of T2D complications found that the cumulative incidence of CVD increases with age in the T2D population [30], the higher prevalence of CVD risk in late-onset T2D may be because age as a risk factor for CVD risk is more pronounced in lat-onset patients in our study. Regarding medication use patterns, the results of a case‒control study indicated that there was a higher rate of antihypertensive medication use at diagnosis in late-onset T2D [31], which is consistent with our result in which patients with late-onset T2D had a higher rate of hypertension prevalence. Furthermore, until 2019, metformin and insulin were the drugs most commonly used by young people with early-onset T2D [33], and our study found a higher use of these two drugs in patients with early-onset T2D. A cohort study of 11,244 patients revealed that nearly half of T2D patients began treatment with sulfonylureas [34], and the most commonly used oral hypoglycemic drug in our patients at initial diagnosis was also sulfonylureas; however, metformin was the most commonly used drug throughout the follow-up period. These results all indicate more severe metabolic disturbances in early-onset patients. The metabolic disorders described above may be one of the reasons why people with early-onset T2D develop hypertension and hyperlipidemia more quickly.

Persistent hyperglycemia is another critical factor. Aliki-Eleni Farmaki et al. [35] observed that high blood glucose levels caused by a high-sugar diet were linked to cardiovascular risk factors such as hypertension and hyperlipidemia in a population-based cohort cross-sectional study. In addition, previous studies have confirmed that prolonged higher HbA1c and blood glucose levels are associated with an increased risk of microvascular comorbidities and macrovascular complications [36, 37]. The increased formation of advanced glycation end products within cells is one possible mechanism [38]. In our study, we discovered that patients with early-onset T2D had higher HbA1c levels and higher random blood glucose levels than patients with late-onset T2D, both at the onset and throughout the course of the study, with the former being especially high at the onset.

One potential reason for complications may be due to poor dietary habits and sedentary lifestyles of young people may also be the cause of complications [31, 39]. As prior studies show, among early-onset T2D patients, unhealthy diet, smoking, irregular physical activity and overweight were more common. According to the scoping review by Ran Bao et al., some researchers reported sedentary time of more than 6 h in young people than in older people [40]. In addition, a higher proportion of early-onset T2D patients may have bad habits such as smoking and drinking than late-onset T2D patients, and early-onset T2D patients bear an even greater psychological burden of stress. An unhealthy diet may lead to obesity, and obesity is known to be a major risk factor for early-onset diabetes, with 80–92% of the population with T2D being obese, compared to 56% of the elderly population [41, 42]. These situations may explain the faster progression of hypertension and hyperlipidemia in early-onset patients in our study [43, 44].

### Strengths and limitations

One strength of our study is the use of a large population-based regional database, including nearly 25 million patients in Tianjin, and all visits in hospitals were linked. Another strength is that for unclassified diabetes, the inclusion and exclusion criteria were applied to strictly screen out patients aged > 30 years at first diagnosis and select patients using only oral hypoglycemic agents in one hospitalization or two outpatient visits as having type 2 diabetes, reducing misclassification. Moreover, we evaluated the clinical characteristics of newly diagnosed patients, which can address the unclear order of occurrence of early-onset T2D and comorbid complications.

Our study also has some limitations. First, our study population consisted of type 2 diabetes patients from Tianjin, which is only representative of northern China. Second, some indicators, such as BMI and C peptide, are missing in our data, which may lead to the classification of a small number of type 1 diabetic patients as type 2 diabetics. Third, we only have data for a 4-year period from 2015 to 2019 with a short follow-up period; thus, there may be some patients who have not yet had an outcome event, resulting in censoring and underestimation of the incidence of comorbid complications of T2D.

## Conclusion

Patients with newly diagnosed early-onset T2D had earlier comorbidities of hypertension and hyperlipidemia, and metabolic disturbance was more severe in patients with early-onset type 2 diabetes. More research is needed to understand its pathophysiologic mechanisms. As early-onset patients may face the threat of other comorbidities, which can greatly affect quality of life, it is crucial to promote healthy diets or lifestyles to prevent T2D onset in young adults and slow the progression of comorbidities.

### Electronic supplementary material

Below is the link to the electronic supplementary material.

Supplementary Material 1

Supplementary Material 2

## Data Availability

The datasets used and analyzed during the current study are available from the corresponding author on reasonable request.
