# Supplementary material for: Comparison of clinical characteristics and disease burden between early- and late-onset type 2 diabetes patients: a population-based cohort study
Source: BMC Public Health. 2023 Dec 4;23:2411. doi: 10.1186/s12889-023-17280-5 (PMC10696789; doi:10.1186/s12889-023-17280-5)
Supplement: Supplementary file 1 — Supplementary Material 1 [file 12889_2023_17280_MOESM1_ESM.docx]

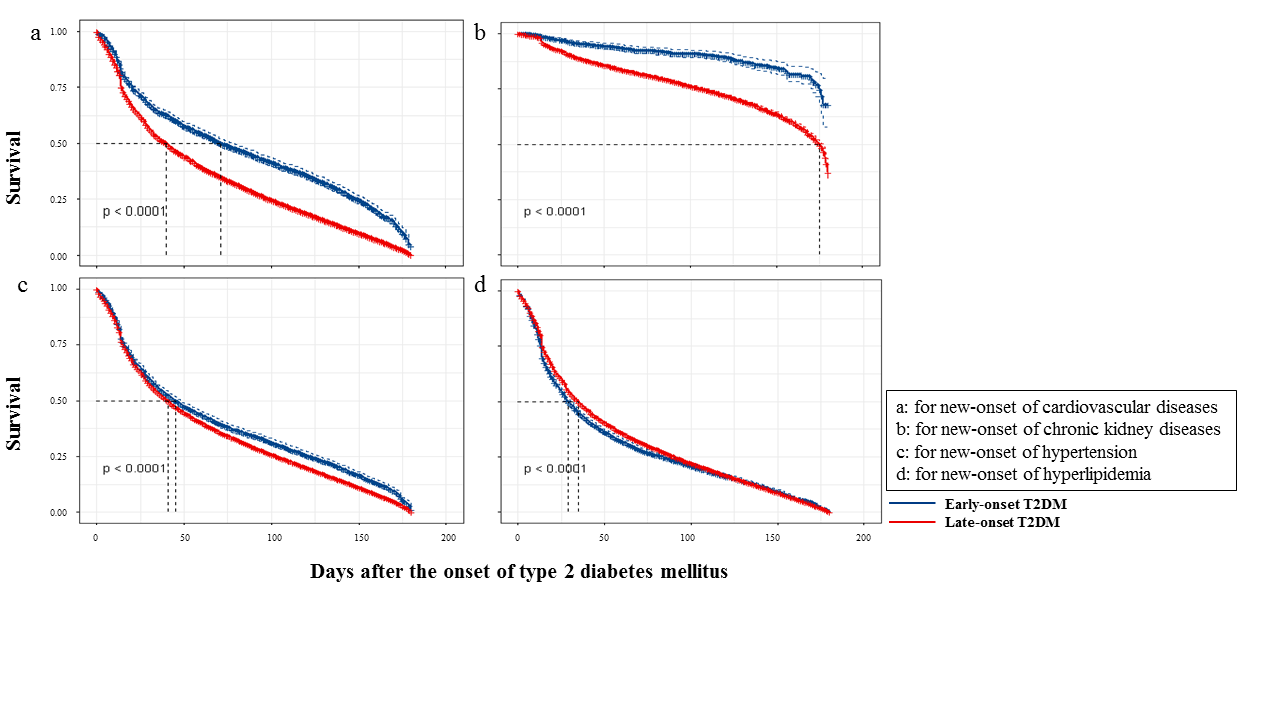


Supplementary Figure 1. Cumulative hazard of developing comorbidities/complications during 180 days after the T2DM onset


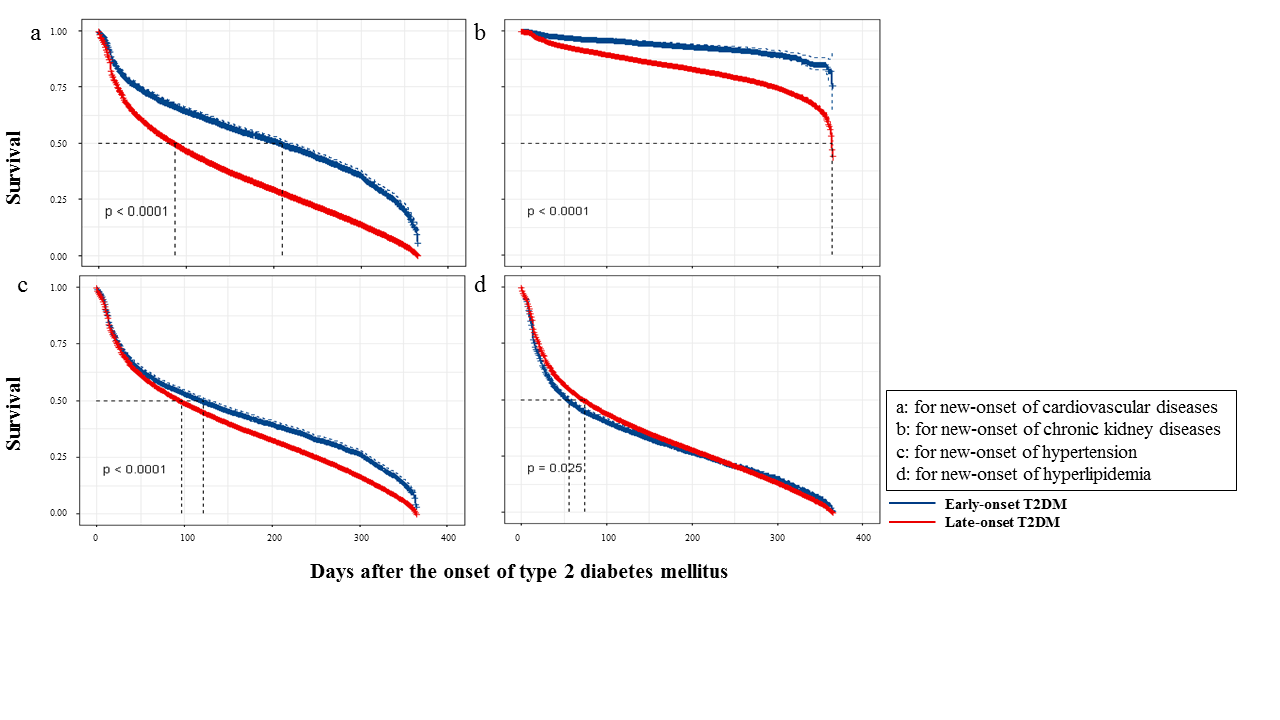


Supplementary Figure 2. Cumulative hazard of developing comorbidities/complications during 365 days after the T2DM onset
