# Supplementary material for: Comparison of clinical characteristics and disease burden between early- and late-onset type 2 diabetes patients: a population-based cohort study
Source: BMC Public Health. 2023 Dec 4;23:2411. doi: 10.1186/s12889-023-17280-5 (PMC10696789; doi:10.1186/s12889-023-17280-5)
Supplement: Supplementary file 2 — Supplementary Material 2 [file 12889_2023_17280_MOESM2_ESM.docx]

Supplementary Tables

Supplementary Table 1. Laboratory measurements of T2D patients of last visit during period (M, IQR)

|  | **T2D**  **(N=652,290)** | **New-onset T2D (N = 322,030)** | | **P value** |
| --- | --- | --- | --- | --- |
|  |  | **Early-onset T2D**  **(N=21,347)** | **Late-onset T2D**  **(N=300,676)** |  |
| **Laboratory measurements (M, IQR)** | | | | |
| HbA1c (%) | 7.3 [6.5, 8.6] | 8.6 [6.9, 10.3] | 7.1 [6.4, 8.3] | <0.001 |
| Random blood glucose (mmol/L) | 7.7 [6.2, 10.3] | 8.7 [6.5, 12.2] | 7.5 [6.1, 9.8] | <0.001 |
| Serum creatinine (μmol/L) | 67.5 [55.0, 86.7] | 115.2 [87.0, 125.6] | 69.4 [51.1, 90.6] | <0.001 |
| eGFR (ml/min/1.73m^2^) | 69.4 [50.3, 92.5] | 102.8 [82.1, 120.0] | 76.0 [58.7, 94.8] | <0.001 |
| MALB (g/24h) | 12.4 [5.8, 45.0] | 13.3 [7.0, 34.2] | 11.5 [5.2, 40.4] | <0.001 |
| HDL (mmol/L) | 1.0 [0.8, 1.2] | 0.9 [0.8, 1.1] | 1.0 [0.8, 1.2] | <0.001 |
| LDL (mmol/L) | 2.7 [2.1, 3.4] | 3.0 [2.3, 3.7] | 2.7 [2.1, 3.4] | <0.001 |
| TG (mmol/L) | 1.4 [1.0, 2.1] | 2.2 [1.4, 3.5] | 1.4 [1.0, 2.0] | <0.001 |
| TC (mmol/L) | 4.3 [3.5, 5.2] | 4.8 [4.0, 5.7] | 4.3 [3.5, 5.2] | <0.001 |

Supplementary Table 2. Laboratory measurements of T2D patients (Mean, SD)

|  | **T2D**  **(N=652,290)** | **New-onset T2D (N = 322,030)** | | **P value** |
| --- | --- | --- | --- | --- |
|  |  | **Early-onset T2D**  **(N=21,347)** | **Late-onset T2D**  **(N=300,676)** |  |
| **During T2D period** | | | | |
| HbA1c (%) | 8.1 (1.7) | 8.3 (2.0) | 7.8 (1.6) | <0.001 |
| Random blood glucose (mmol/L) | 9.2 (3.3) | 9.1 (3.3) | 8.8 (3.0) | <0.001 |
| Serum creatinine (μmol/L) | 76.9 (67.7) | 71.7 (71.1) | 75.5 (61.2) | <0.001 |
| eGFR (ml/min/1.73m^2^) | 75.4 (27.1) | 97.4 (32.0) | 75.1 (24.9) | <0.001 |
| MALB (g/24h) | 87.1 (375.1) | 67.8 (190.2) | 73.8 (287.1) | 0.548 |
| HDL (mmol/L) | 1.2 (0.3) | 1.1 (0.3) | 1.2 (0.3) | <0.001 |
| LDL (mmol/L) | 3.1 (0.9) | 3.1 (0.9) | 3.0 (0.9) | <0.001 |
| TG (mmol/L) | 1.9 (1.5) | 2.7 (2.4) | 1.8 (1.2) | <0.001 |
| TC (mmol/L) | 4.9 (1.2) | 5.0 (1.2) | 4.8 (1.2) | <0.001 |
| **Last visit during T2D period** |  |  |  |  |
| HbA1c (%) | 7.7 (1.7) | 8.7 (2.2) | 7.5 (1.6) | <0.001 |
| Random blood glucose (mmol/L) | 8.8 (3.9) | 9.8 (4.5) | 8.4 (3.6) | <0.001 |
| Serum creatinine (μmol/L) | 90.1 (95.8) | 80.1 (110.3) | 86.3 (84.3) | 0.061 |
| eGFR (ml/min/1.73m^2^) | 70.2 (28.7) | 100.7 (33.7) | 69.4 (26.4) | <0.001 |
| MALB (g/24h) | 162.5 (667.2) | 120.7 (498.9) | 150.9 (690.2) | 0.617 |
| HDL (mmol/L) | 1.1 (0.3) | 1.0 (0.3) | 1.1 (0.3) | <0.001 |
| LDL (mmol/L) | 2.8 (1.0) | 3.0 (1.1) | 2.8 (1.0) | <0.001 |
| TG (mmol/L) | 1.8 (1.5) | 3.2 (3.3) | 1.7 (1.3) | <0.001 |
| TC (mmol/L) | 4.5 (1.4) | 5.1 (1.8) | 4.4 (1.3) | <0.001 |
